# Synaptotoxic effects of extracellular tau are mediated by its microtubule-binding region

**DOI:** 10.1007/s00401-025-02897-0

**Published:** 2025-06-02

**Authors:** Tomas Ondrejcak, Neng-Wei Hu, Emily Coode, Tom Campbell, Grant T. Corbett, Ivan Doykov, Kevin Mills, Dominic M. Walsh, Frederick J. Livesey, Michael J. Rowan, Igor Klyubin

**Affiliations:** 1 Department of Pharmacology and Therapeutics, School of Medicine, and Institute of Neuroscience, Trinity College, Watts Building, Dublin 2, Ireland; 2 Department of Physiology and Neurobiology, School of Basic Medical Sciences, Zhengzhou University, 100 Science Avenue, Zhengzhou, 450001 China; 3 Talisman Therapeutics, Babraham Research Campus, Cambridge, CB22 3AT UK; 4 Laboratory for Neurodegenerative Research, Brigham and Women’s Hospital and Harvard, Ann Romney Center for Neurologic Diseases, Medical School, Boston, MA USA; 5 Translational Mass Spectrometry Research Group, University College London Institute of Child Health and Great Ormond Street Hospital, London, UK; 6 Gen2 Neuroscience, Babraham Research Campus, Cambridge, CB22 3AT UK

**Keywords:** Long-term potentiation, Tauopathies, Mass spectrometry, Hippocampus, In vivo

## Abstract

Immunotherapies targeting extracellular tau share the premise that interrupting cell-to-cell spread of tau pathology in Alzheimer’s disease (AD) will slow dementia pathogenesis. Whether these interventions affect the actions of synaptotoxic, extracellular tau species that may contribute to cognitive impairment is relatively unknown. Here, we assayed synaptic plasticity disruption in anaesthetised live rats caused by intracerebral injection of synaptotoxic tau present either in (a) secretomes of induced pluripotent stem cell-derived neurons (iNs) from people with Trisomy 21, the most common genetic cause of AD, or (b) aqueous extracts of human AD brain. Extracellular tau in iN secretomes was found to include fragments that contain the extended microtubule-binding regions of tau, MTBR/R’ and adjacent C-terminal sequences. Immunodepletion or co-injection with antibodies targeting epitopes within these fragments prevented the acute disruption of synaptic plasticity by these patient-derived synaptotoxic tau preparations. Moreover, a recombinant human tau fragment encompassing the core MTBR/R’-region present in tau fibrils, tau_297-391_, potently mimicked the deleterious action of patient-derived tau. MTBR/R’-directed antibodies also rapidly reversed a very persistent synaptotoxic effect of soluble brain tau. Our findings reveal a hitherto relatively unexplored potential benefit of targeting extracellular MTBR/R’ tau on correcting synaptic dysfunction.

**Supplementary Information:**

The online version contains supplementary material available at 10.1007/s00401-025-02897-0.

## Introduction

Although great strides have been made in understanding the physiology and pathology of the microtubule-associated protein, tau, we still lack successful tau-based therapeutics. Some of the most advanced tau-targeting trial therapies for Alzheimer’s disease (AD) are based on use of antibodies intended to bind and neutralise extracellular tau species capable of propagating tau pathology. Besides the potential spreading of tau aggregation between neurons, there is evidence that certain forms of extracellular tau have pathogenic properties, including synaptic plasticity-disrupting activity [28, 29].

In human brain there are six different splice isoforms of tau that give rise to proteins of 352–441 amino acids, all of which undergo posttranslational modifications including truncation. In general, full-length tau and C-terminal (CT) fragments are mainly found inside, whereas N-terminal (NT) and mid-region (MR) fragments predominate outside neurons under healthy conditions [1, 24, 31, 46, 50]. Only fragments which contain the microtubule-binding region (MTBR) are competent to aggregate [12, 58, 59]. Clinical trials targeting the NT of tau have been unsuccessful and focus has shifted to testing antibodies that target epitopes in or near the MTBR. Tau fragments that include the MTBR and the CT adjacent pseudorepeat R’(aa369-399) [19] (referred to as the MTBR/R’) are enriched in tauopathy brain, including AD [2, 56]. Indeed, the accumulation of certain MTBR fragments in the brain is correlated with cognitive impairment [2, 26, 56], and levels of specific MTBR peptides in CSF correlate with tau tangle load within the CNS [26], providing support for investigating MTBR-targeting strategies. More detailed understanding of the role of soluble extracellular MTBR-containing tau fragments in AD should inform planned clinical trials.

AD is characterised by synaptic failure, but little is known about the vulnerability of synapses to MTBR-containing tau fragments. Several preparations are available to investigate the synaptotoxicity of tau including recombinant tau, patient-derived brain samples, and secretomes from induced pluripotent stem cell (iPSC)-derived neurons (iN). Previously, we reported that removing tau from aqueous brain extracts of some AD patients prevented inhibition of long-term potentiation (LTP), an electrophysiological correlate of memory [40–42]. In addition, we discovered that the secretomes of iNs from people with Down syndrome (Trisomy 21, Ts21), the most common cause of early onset AD [36], also disrupt synaptic plasticity in a tau-dependent manner [28]. Both patient-derived preparations contain a range of tau species. Secretomes from iNs, like human CSF, have abundant NT and MR fragments of tau with low levels of MTBR-containing tau [8, 18, 28, 50], whereas aqueous extracts of AD brain contain mostly intracellular full-length and MTBR-containing tau [50].

Here, we studied the involvement of extended MTBR/R’-containing tau species [12] in the acute inhibition of LTP by patient-derived soluble synaptotoxic tau in anaesthetised rats in vivo. We also tested the ability of anti-tau antibodies targeting MTBR/R’ (Gen2B) and the adjacent CT region (Gen2A) to reverse a persistent disruption of synaptic plasticity induced by tau-containing AD brain extracts.

## Materials and methods

### Generation of cortical cultures and secretome preparation

The iPSC lines used in this study were non-demented control (NDC) [30] and Ts21 [43]. Ts21 iPSCs were generated from two individuals with Down syndrome (referred to as Line 1 and Line 2) using the CytoTune-iPS 2.0 Sendai Reprogramming Kit (ThermoFisher). Human cortical neurons were produced from all iPSC lines essentially as described [51–53].

**Fig. 1:**
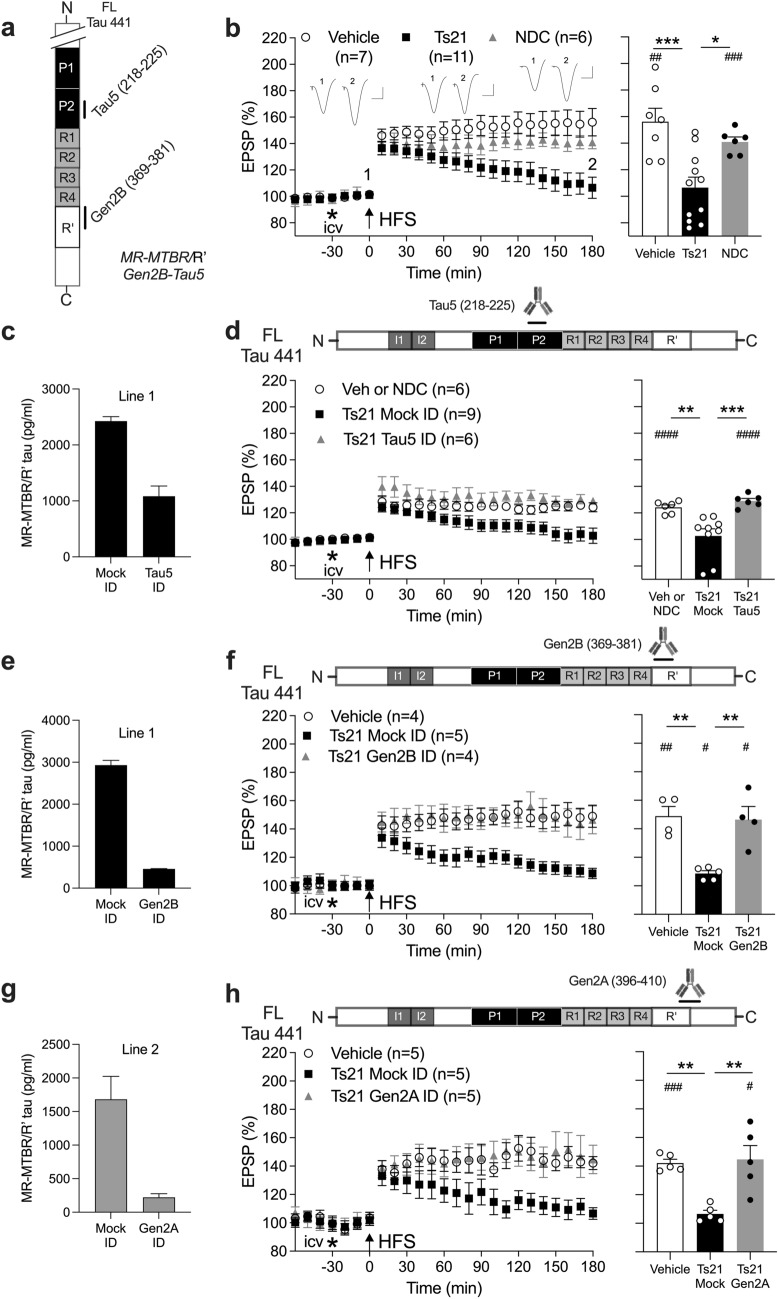
MTBR/R’-containing extracellular tau fragments secreted by Ts21 iNs are synaptotoxic. **a** Antibodies used to capture/detect MR-MTBR/R’ tau fragments in a custom Meso Scale Diagnostics assay (**c**, **e**, and **g**). **b** High-frequency stimulation (HFS, arrow) triggered robust LTP of hippocampal synaptic transmission in anaesthetised (urethane, 1.6 g/kg, i.p.) rats after intracerebroventricular (i.c.v, asterisk) injection of 15–20 µL of either vehicle or the secretome from non-demented control (NDC) iPSC-derived iNs. In contrast, LTP decayed to baseline 3 h after application of HFS in animals injected with secretomes from one of two Ts21 lines. **c** Immunodepletion (ID) with the monoclonal MR-directed antibody Tau5 lowered MTBR/R′ tau level by ~ 55%. **d** After ID with Tau5, Ts21 secretomes no longer inhibited LTP. **e–h** ID either with the monoclonal antibody Gen2B, directed at R’, (**e** and **f**), or Gen2A, directed at R’ and the adjacent CT, (**g** and **h**), caused a > 85% reduction of MR-MTBR/R’ fragment concentration, and prevented the inhibition of LTP by Ts21 secretomes. In **b, d, f** and **h**, left-hand panels show the time course of LTP. Summary bar charts of LTP magnitude during the last 10 min are displayed in the right-hand panels. Insets in **b** show representative field EPSP traces at the times indicated. Calibration bars: vertical, 1 mV; horizontal, 10 ms. In **b, d, f** and **h**, values are mean ± SEM. ^#^p < 0.05, ^##^p < 0.01, ^###^*p* < 0.001, ^####^*p* < 0.0001 compared with pre-HFS, paired *t*-test; *p < 0.05, **p < 0.01, ***p < 0.001, one-way ANOVA followed by Bonferroni’s multiple-comparison tests

**Fig. 2:**
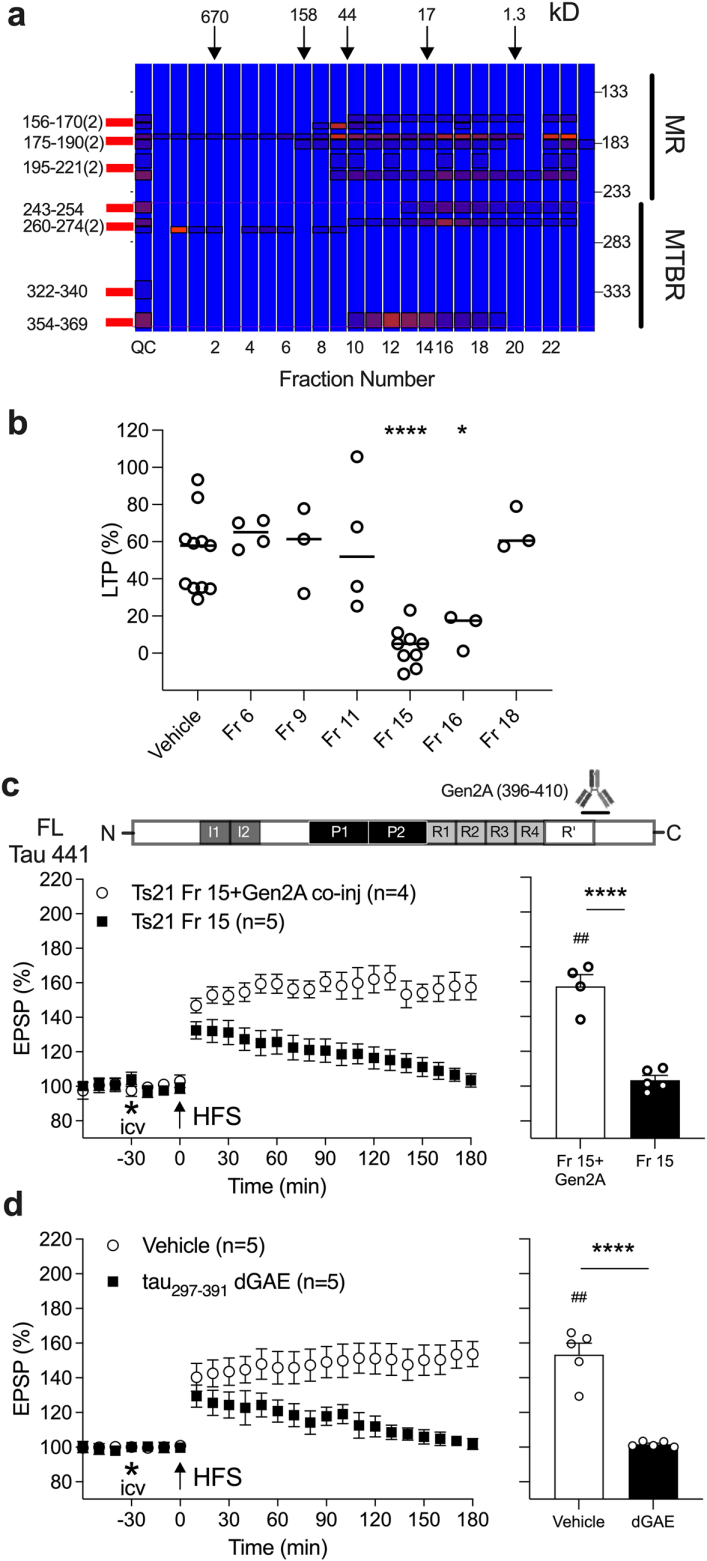
Tau fragments in certain Ts21 iN secretome size exclusion chromatography (SEC) fractions potently inhibit LTP in an MTBR/R’-CT-dependent manner. **a** Mass spectrometry (MS) analysis of MR and MTBR tau in SEC-fractionated Ts21 iN secretomes. Tau fragments containing MTBR appear to be most abundant in fractions 12–14 and 16–17. Note that insufficient quantity of fraction 15 was available following the functional LTP experiments to analyse by MS. The elution of globular protein standards is indicated by downward pointing arrows. An increase in red colour intensity denotes an increase in peptide abundance. Quality control (QC) sample comprises all fractions analysed (see Fig. 5S). Unique 2N4R tau peptide sequences used in the targeted assay are shown on the left (see also Table 2S). Data from adjacent fragments containing unique tryptic peptides were combined (2). MR and MTBR tau are specified on the right. **b** In anaesthetised rats, i.c.v. injection of 20 µL of Ts21 Mock ID SEC fractions 15 (Fr 15) and 16 (Fr 16) significantly inhibited hippocampal LTP. Circles represent individual animal values. *p < 0.05, ****p < 0.0001, compared with Vehicle group, one-way ANOVA followed by Bonferroni’s multiple-comparison tests. **c** Co-injection of Gen2A mAb (2.5 µg per injection) prevented the inhibition of LTP by Fr 15 (10 µL of 2X concentrated Fr 15). **d** The recombinant human tau fragment tau_297-391_ (also known as dGAE fragment [39]) (80 pg/injection in 5 µL i.c.v.) potently inhibited LTP. This dose did not affect baseline synaptic transmission in the absence of HFS conditioning stimulation (pre- vs 210 min post-injection, n = 3, p = 0.3778, data not shown). Importantly, the amount of tau_297-391_ used was similar to MR-MTBR/R’-containing fragments in the Ts21 unfractionated secretomes (34–60 pg/injection i.c.v., Fig. 1c,e,g). Left-hand panels in **c, d** show the time course of LTP. Summary bar charts of LTP magnitude during the last 10 min are displayed in the right-hand panels. Values are mean ± SEM. ^##^*p* < 0.01, compared with pre-HFS, paired *t*-test; ****p < 0.0001, unpaired *t*-test tests

**Fig. 3:**
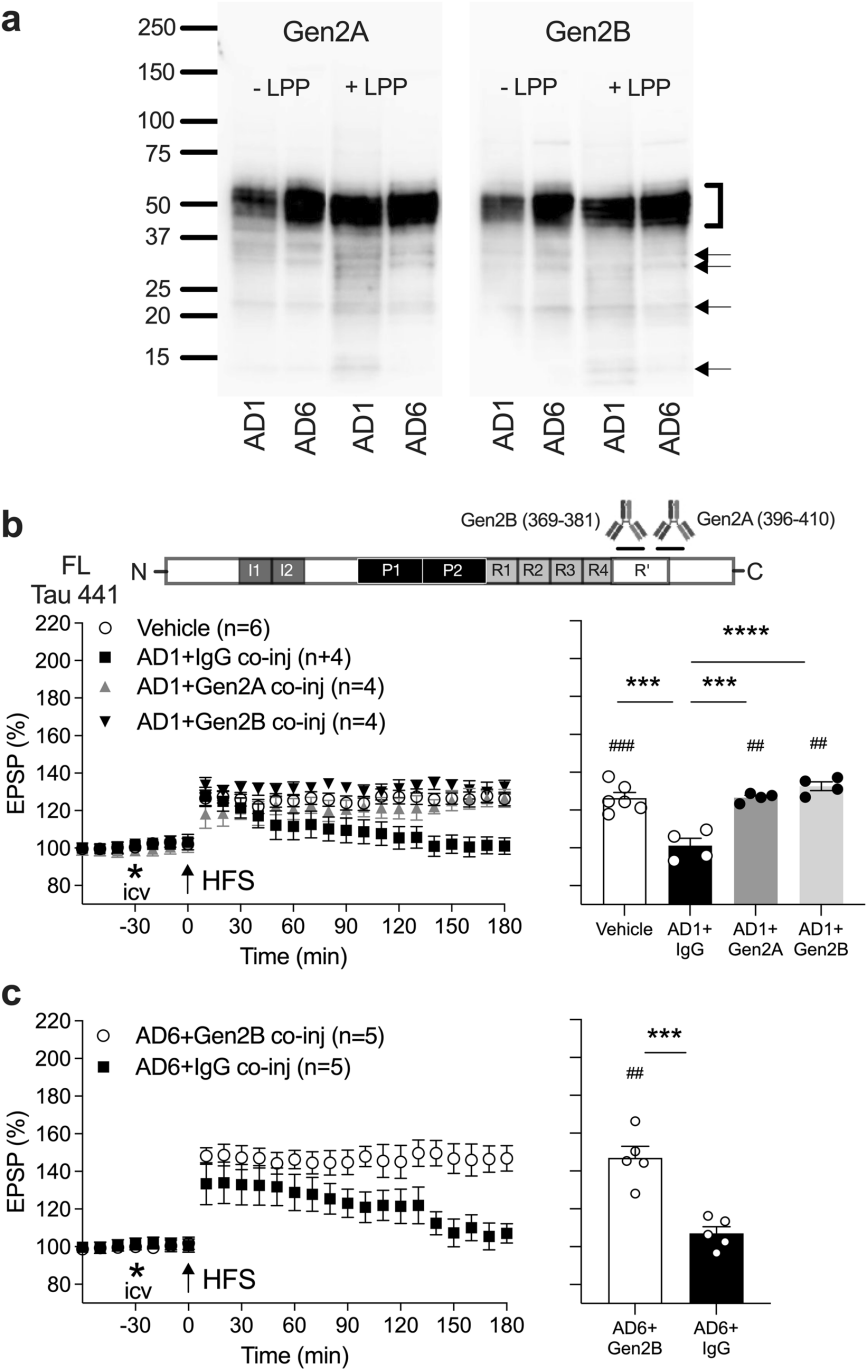
MTBR/R’ is required for acute plasticity disruption by synaptotoxic tau in AD brain extracts. **a** Aqueous extracts from the brains of two people with AD (AD1 and AD6) were analysed by Western blotting in the presence or absence of lambda protein phosphatase (± LPP) using the monoclonal anti-tau antibodies Gen2A and Gen2B. Migration of SDS-PAGE molecular weight standards (in kDa) is indicated on the left. The bracket on the right highlights the position of full-length tau whereas arrows indicate likely tau fragments detected by both mAbs. It should be noted that AD6 extract had much higher total tau concentration as measured by immunoassay [41]. **b** Unlike isotype control IgG, anaesthetised rats co-injected with AD1 extract (10 µL) and either Gen2A or Gen2B antibodies (2.5 µg i.c.v.) maintained normal hippocampal LTP. **c** Similarly, robust LTP was induced when Gen2B mAb (2.5 µg i.c.v.) was co-administered with the extract AD6 (10 µL). Left-hand panels in **b, c** show the time course of LTP. Summary bar charts of LTP magnitude during the last 10 min are displayed in the right-hand panels. Values are mean ± SEM. ^##^*p* < 0.01, ^###^*p* < 0.001 compared with pre-HFS, paired *t*-test; ***p < 0.001, ****p < 0.0001, one-way ANOVA followed by Bonferroni’s multiple-comparison tests in **b** and unpaired *t*-test in **c**

Human iPSCs were maintained on Geltrex in Essential 8 media (both ThermoFisher). Secretomes of differentiated neurons were collected between day 70 and day 80 post-neural induction at 48 h intervals, aliquoted and stored in protein lo-bind tubes at − 80 °C. The majority of secretome preparations (Figs. 1b,d, 2a-c; Figs. 1S, and 3S) were cleared of cell debris and extracellular vesicles by centrifugation. First, the secretome was centrifuged at 200×*g* and 4 °C for 10 min. Then, the upper 97% was recovered and centrifuged at 2000×*g* and 4 °C for 10 min. Next, the upper 97% of this was recovered and centrifuged at 10,000×*g* and 4 °C for 30 min. Finally, the upper 97% was recovered and centrifuged at 100,000×*g* and 4 °C for 70 min.

All secretomes were dialyzed (using Slide-A-Lyzer™ G2 Dialysis Cassettes, 2 K MWCO, ThermoFisher) against artificial CSF (aCSF; 124 mM NaCl, 2.8 mM KCl, 1.25 mM NaH2PO4, 26 mM NaHCO3) to remove bioactive small molecules. Dialysis was performed at 4 °C against a 100-fold excess of aCSF with buffer changed three times over a 48-h period. Dialyzed secretome was either frozen at − 80 °C or used immediately for further biochemical manipulations.

### Human brain tissue

Frozen tissue was obtained from two cases of end-stage AD (referred to as AD1 and AD6) (see Table 1 in Ondrejcak et al*.*, [40]). Brain sample from AD1 was obtained from the Massachusetts Alzheimer’s Disease Research Center Neuropathology Core at Massachusetts General Hospital. AD6 brain was from the Banner Health brain bank. Aqueous brain extracts were prepared from cortical grey matter by centrifugation and dialysis (2 K molecular weight cutoff) of 20% (weight/volume) homogenates, as described in our previous publications [40, 41].

### Immunodepletion (ID)

Ts21 secretomes underwent 1–3 rounds of 12–16 h ID by incubation with Protein A Sepharose beads (PAS; 10 μL, ThermoFisher) at 4 °C with either (i) anti-tau antibodies (Tau5, Tau46, K9JA, Gen2A or Gen2B) and/or (ii) control antibodies i.e. 46–4 or rabbit IgG, or purified pre-immune serum (Mock). In each case, samples were cleared of beads and then both the ID and ‘mock’ ID samples were incubated with PAS alone to remove previously unbound IgG. ID supernatants were stored at -80 °C.

**Fig. 4:**
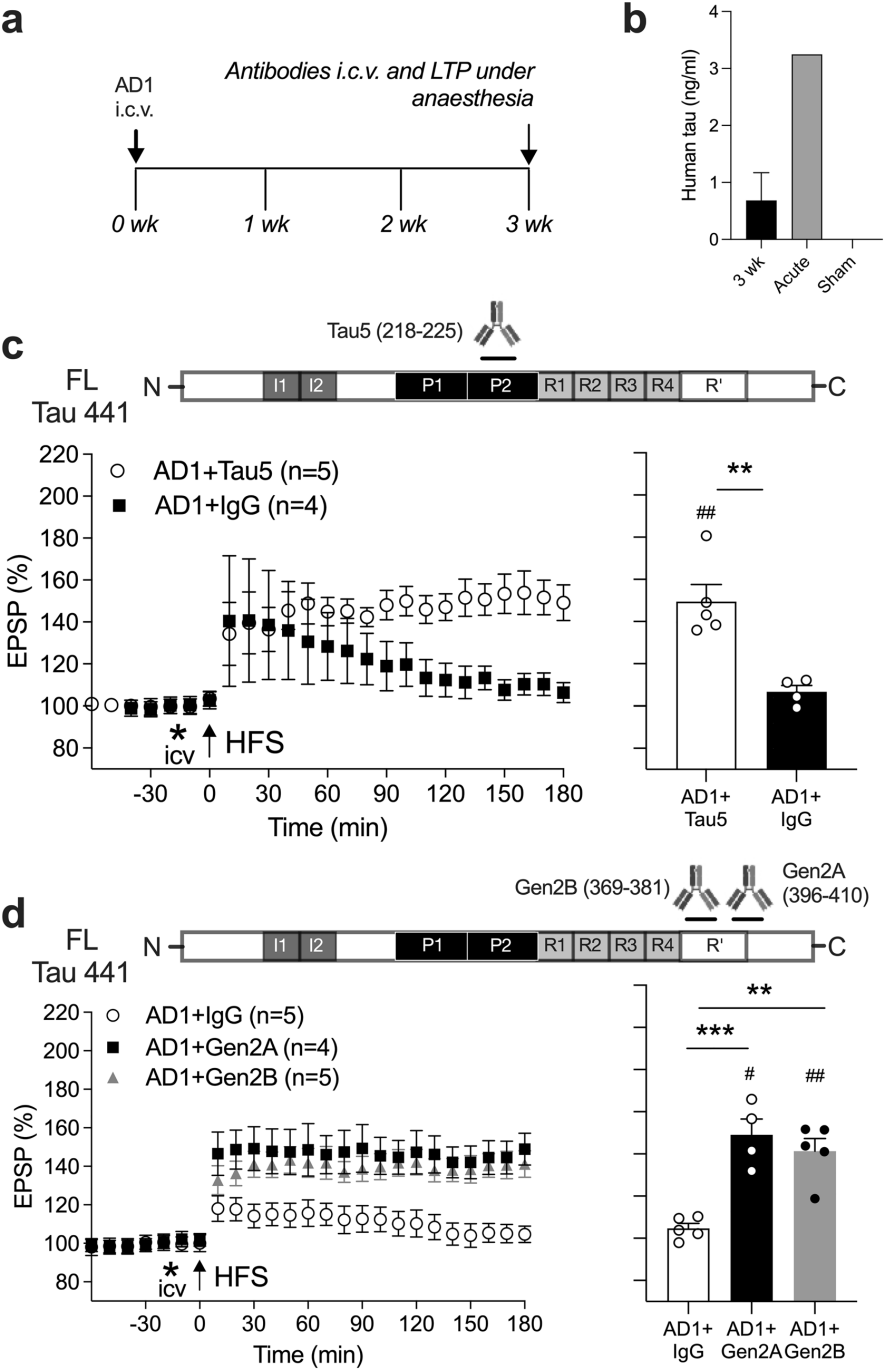
Rapid reversal of the persistent inhibition of LTP by synaptotoxic tau in AD aqueous brain extract by acute injection of anti-tau antibodies. **a** Study design. Animals received an i.c.v. injection of AD1 extract under recovery anaesthesia (ketamine and medetomidine, 60 and 0.4 mg/kg, respectively, i.p.), followed 3 weeks later by a single injection with anti-tau or control antibodies under non-recovery urethane anaesthesia. **b** Human tau was detected in rat hippocampus 3 weeks (3 wk, n = 18, after i.c.v. injection of 10 µL of AD1). LLoQ = 0.03125 ng/ml (see Fig. 4S). For comparison, the levels of human tau detected after acute (30 min) injection with the same volume of AD1 (pooled sample from 3 rats) or a sham injection (n = 1) are also shown. **c** Whereas LTP was strongly inhibited in animals receiving control mAb IgG (AD1 + IgG), robust LTP was induced in rats injected with the mid-region anti-tau mAb Tau5 (AD1 + Tau5) (2.5 μg i.c.v., 15 min prior to HFS). **d** The same protocol was followed using anti-tau mAbs targeting the MTBR/R’ or MTBR/R’ and the adjacent CT region of tau with Gen2B (AD1 + Gen2B) or Gen2A (AD1 + Gen2A), respectively. HFS triggered stable LTP in these animals. Left-hand panels in **c, d** show the time course of LTP. Summary bar charts of LTP magnitude during the last 10 min are displayed in the right-hand panels. Values are mean ± SEM. ^#^*p* < 0.05, ^##^*p* < 0.01 compared with pre-HFS, paired *t*-test; **p < 0.01, ***p < 0.001 unpaired *t*-test in **c** and one-way ANOVA followed by Bonferroni’s multiple-comparison tests in **d**

To rule out possible involvement of amyloid beta (Aß), in some cases, we used Ts21 secretomes (Fig. 1Sb) and AD1 extract (Figs. 3a, 4b, d, and Fig. 4S) that had been previously subjected to ID treatment with the polyclonal anti-Aß antibody AW7 [28, 41].

### Tau enzyme-linked immunosorbent assays (ELISAs)

Assays for MR and CT tau in Ts21 secretomes were performed using specific combinations of capture and detection antibodies [40–42]. For all assays, the capture antibody was coated at 2.5 μg/ml in Tris-buffered saline (TBS) for 1 h at 37 °C and 300 rpm agitation. Plates were then washed three times with 100 μL TBS with Tween-20 (TBST) prior to blocking in 100 μL TBS containing 3% BSA for 2 h at RT and 300 rpm. Plates were washed three times with 100 μL TBST before 25 μL of samples (diluted 1:1 in TBS containing 1% BSA) and standards were applied in triplicate and agitated for 16 h at 4 °C. Importantly, the same calibration standard (recombinant human tau441) was used for all assays, thus enabling comparison of concentrations detected by different assays. The following day, 25 μL alkaline phosphatase conjugated detection antibodies diluted 1:250 in TBST containing 1% BSA were added directly to the plates without washing and incubated for 1 h at RT and 300 rpm agitation. Finally, plates were washed three times with 100 μL TBST before 50 μL Tropix Sapphire II (Applied Biosystems) detection reagent was added and incubated for 30 min at RT and 300 rpm agitation. Standard curves were fitted to a five-parameter logistic function with 1/Y2 weighting using MasterPlex ReaderFit (MiraiBio).

### Custom MSD (Meso Scale Diagnostics) assay for tau containing the MTBR/R’ domain

To quantify extracellular levels of MTBR/R'-containing tau fragments, a custom MSD electrochemiluminescence assay was developed using uncoated MULTI-ARRAY 96-well SECTOR plates (MSD), Gen2B (epitope in aa369-381 of 2N4R tau) and Tau5 (epitope in aa218-225) antibodies (Fig. 1a).

All solutions were prepared in PBS, supplemented with 5% BSA for blocking solution, 1% BSA for detection antibody solution, or 0.05% Tween-20 for wash solution. Plates were coated overnight with 4 µg/ml monoclonal rabbit anti-tau (aa369-375) capture antibody (Gen2B), followed by blocking for 1 h. Conditioned media samples or recombinant full-length monomeric tau (rPeptide, T-1001) were incubated for 3 h. For detection, plates were incubated overnight with 2 µg/ml monoclonal Tau5 mouse anti-tau (aa218-225) detection antibody (ThermoFisher Scientific, AHB0042) and detected with 1 µg/ml goat anti-mouse SULFO-TAG secondary antibody (MSD, R32AC) for 1 h. Signal was generated using MSD Gold Read Buffer B and detected by MESO QuickPlex SQ 120MM Imager (MSD). Concentrations of MTBR/R’-containing tau in conditioned media samples were calculated with reference to the standard curve using MSD Discovery Workbench software.

### Size exclusion chromatography (SEC)

Twelve millilitre of aliquots of ultracentrifuged and dialyzed secretomes was concentrated tenfold (to 1.2 ml) using Amicon Ultra-15 3 kDa centrifugal filters (Millipore, Billerica, MA) at 4 ºC. Immediately thereafter, 1 ml of concentrate was chromatographed on tandem Superdex 200 Increase–Superdex 75 10/300 GL (GE, Marlborough, MA) columns eluted in 50 mM ammonium bicarbonate (pH 8.5) at a flow rate of 0.5 ml/min using Pharmacia FPLC system (Amersham Life Sciences, Uppsala, Sweden). One millilitre of the fractions were collected. SEC was repeated twice using both Ts21 Line 1 and Line 2. A 900 μL of each fraction was lyophilised, and the remaining volume was aliquoted and stored at -80 ºC pending analyses using tau immunoassays and electrophysiology. Since it was necessary to concentrate conditioned media prior to SEC, we were careful to confirm that the concentration step did not remove synaptotoxic activity. Similar to unconcentrated media, concentrated Ts21 secretomes inhibited LTP in vivo, and this inhibition was prevented by Tau5 ID (Ts21 Mock ID, n = 4, vs Ts21 Tau5 ID, n = 4, p = 0.012, data not shown).

### Mass spectrometry (MS)

Liquid chromatography with tandem MS analysis, similar to that described previously [11], was used to investigate MTBR-containing tau SEC fractions of iN secretomes. Please refer to Supplementary materials for full details.

### Western immunoblot analysis

Aqueous extracts from the brains of two people with AD (AD1 and AD6) were analysed for tau protein by western immunoblotting in the presence or absence of lambda protein phosphatase (LPP) concentrate supplied at 400,000 units/ml, diluted 1:50 to 8000 units/ml for final reaction as per kit recommendation (New England Biolabs P0753S). Samples were run on 4–15% Criterion TGX Stain-Free Protein Gels (Bio-Rad) and proteins transferred to Trans-Blot Turbo Midi 0.2 µm PVDF membranes (Bio-Rad). Membranes were blocked with 5% BSA in TBST for 1 h at RT before an overnight incubation with primary antibodies (monoclonal Gen2A rabbit anti-tau IgG, 1:1000; monoclonal Gen2B rabbit anti-tau IgG, 1:1000) in blocking solution at RT. For detection, membranes were washed in TBST following primary antibody incubation and then incubated with fluorescent secondary antibodies (goat anti-rabbit-647 IgG, 1:2000, ThermoFisher Scientific A21244) in blocking solution for 2 h at RT, washed in TBST and imaged in the 647 and 568 channels on a ChemiDoc Imaging System (Bio-Rad).

### Antibodies

The antibodies used and their sources are described in Table 1S. The monoclonal rabbit antibodies Gen2A and Gen2B were generated and characterised extensively as described previously [32, 33].

### Compounds

Tau_297-391_, also known as dGAE, the truncated recombinant human tau fragment encompassing the core MTBR/R’-region present in tau fibrils, was kindly provided by StressMarq Biosciences Inc. (Victoria, Canada) in its monomer form.

### Animals, surgery and electrophysiology

Rats were group-housed, unless otherwise stated. Food and water were available ad libitum with a 12-h light/dark cycle. For most experiments, we used 3–5-month-old male Lister Hooded rats. In the study examining the effect of Gen2A ID (Fig. 1h), we employed older (6–7-month-old) Wistar Han rats. Control experiments were interleaved randomly throughout, and no animals were excluded. Totally, 274 rats were used in this study.

To inject samples and test synaptic plasticity, the animals were anaesthetised with urethane (1.6 g/kg, i.p.) and core body temperature was maintained at 37.5 ± 0.5 °C. An intracerebroventricular (i.c.v) stainless steel guide cannula (22 gauge, 0.7 mm outer diameter, length 13 mm) was implanted above the right lateral ventricle (coordinates, 0.5 mm posterior to bregma and 1.2 mm right of midline, depth 4 mm) before the electrodes were implanted ipsilaterally. Teflon-coated tungsten wire (external diameter 75 µm bipolar or 112 μm monopolar) electrodes were positioned in the stratum radiatum of area CA1. The electrodes were optimally located using a combination of physiological and stereotactic indicators (3.8 mm posterior to bregma and 2.5 mm lateral to midline, and 4.6 mm posterior to bregma and 3.8 mm lateral to midline for recording and stimulating electrodes, respectively). Screw electrodes located over the contralateral cortex were used as reference and earth. To inject samples acutely, a Hamilton syringe was connected to the internal cannula (28 gauge, 0.36 mm outer diameter). The injector was removed 1 min post-injection and a stainless-steel plug was then inserted.

The timing and doses/volumes of i.c.v. injections were based on pilot experiments and our previous experience with these materials and models [28, 40, 41]. An i.c.v. dose of 2.5 µg antibody was chosen based on our previous research when antibodies were injected together with [28, 29, 40] or after [41, 42] patient-derived synaptotoxic tau. This amount of injected antibody is expected to achieve no more than ~ 1 ng/ml in the brain interstitial fluid (ISF) at the time of high-frequency stimulation (HFS) [3, 7, 38, 57, 60]. In co-injection studies, the antibody and tau-containing samples were mixed and immediately injected without pre-incubation together*.* Based on total human tau ELISA kit (Invitrogen, #KHB0041) measurements, the doses of tau in the unfractionated secretome (15–20 µL per i.c.v. injection over 15 min) and AD extracts (10 µL per i.c.v. injection over 5–10 min) are ~ 40 pg/rat and ~ 46 ng/rat, respectively. The concentrations in ISF expected, assuming ISF concentration is approximately half of that in CSF at 30 min post-injection [7, 57], are unlikely to exceed ~ 40 pg/ml and ~ 46 ng/ml total human tau, respectively. The dose of Tau_297-391_ injected i.c.v. (80 pg per rat) is expected to achieve ~ 80 pg/ml in the brain ISF at the time of HFS, based on the same assumptions [7, 57].

We only tested the ability of Gen2A to prevent the disruptive effect of Fraction 15 of Ts21 iN secretomes (Fig. 2c) and Gen 2B against AD6 soluble brain extract (Fig. 3c) to conserve samples and limit the number of experimental animals.

To investigate the persistence of the effects of AD1 brain extract, the i.c.v. injections were administered under recovery anaesthesia using a mixture of ketamine and medetomidine (60 and 0.4 mg/kg, respectively, i.p.), and the cannula was then removed. Afterwards, rats were housed individually in their home cages. Subsequently, electrophysiological recordings were conducted under non-recovery urethane anaesthesia, as described below, 21 days later. An i.c.v. dose of 2.5 µg antibody (in 5 µL over 3 min) was injected 15 min prior to the HFS. At the end of the experiment, both hippocampi were dissected, homogenised and frozen at -80 °C.

To study synaptic plasticity, field excitatory postsynaptic potentials (EPSPs) were evoked and recorded in the stratum radiatum under urethane anaesthesia. Single square-wave pulse (0.2 ms duration) was applied every 30 s and at intensity that triggered a 50% maximum EPSP response. To induce LTP, a 200 Hz HFS protocol consisting of one set of 10 trains of 20 pulses (inter-train interval of 2 s) at test intensity was applied. The magnitude of control LTP varied considerably over the period of carrying out these experiments. To minimise the possible confounding effect of such variation in control LTP, the experiments in each study were interleaved.

For electrophysiological experiments, values are presented as the mean ± SEM of pre-HFS baseline EPSP amplitude over a 30 min period. The magnitude of LTP was measured at 3 h post-HFS and expressed as the mean ± SEM % baseline. For graphing purposes, EPSP amplitude measurements were grouped into 10 min epochs (average of 20 sweeps).

### Rat hippocampal tissue lysate preparation and analysis

After euthanasia, hippocampi were dissected and stored frozen. Immediately prior to analysis, tissue was thawed and homogenised in Pierce™ RIPA buffer (ThermoFisher Scientific, #89900, one part of tissue to 7 parts of lysis buffer) containing protease and phosphatase inhibitor cocktail (Sigma-Aldrich, #P8849, 1 mM final concentration) to ensure protein integrity. Tissue and cell debris was removed by centrifugation (10,000×*g* for 5 min at 4 °C).

To measure human tau in rat hippocampal brain homogenates (Fig. 4b), total human tau ELISA kit (Invitrogen, #KHB0041) was used in accordance with manufacturer’s instructions.

### Data analysis

Electrophysiological data are expressed as the average EPSP amplitude during the last 10 min epoch before and 170–180 min after HFS. Sample sizes were chosen based on our previous publications [40, 41]. The ability to induce LTP within each group was assessed a priori using paired two-tailed *t* tests. Differences in the magnitude of potentiation between groups were analysed using one- or two-way ANOVA with Bonferroni’s post hoc tests or by unpaired two-tailed *t* tests, as appropriate. A *p* value of < 0.05 was considered statistically significant. Statistical analyses were performed in GraphPad Prism software (10.4.1). For immunoassays, bars represent mean of two technical replicates ± SD.

## Results

### MTBR/R’-containing tau fragments in Ts21 iN secretomes mediate inhibition of hippocampal LTP

First, we explored the role of MTBR/R’-containing fragments in the synaptic plasticity-disrupting action of extracellular tau secreted by Ts21 iNs. The most abundant tau fragments in Ts21 secretomes are recognised by Tau5, a monoclonal antibody targeting the MR of tau (aa218-225). The baseline level of MR-containing tau in the Ts21 iN secretomes is ~ 10 ng/ml (Fig. 1S and [28]), which is at the lower end of MR-containing tau concentration range previously reported in tau transgenic mouse ISF using microdialysis [1, 4, 61]. Consistent with our previous report [28], i.c.v. injection of Ts21, but not NDC, iN secretomes robustly inhibited hippocampal LTP in anaesthetised rats (Fig. 1b). Interestingly, using different tau immunoassays (see Fig. 1a and Fig. 1Sa), we found that Tau5 ID not only reduced the MR but also, to a lesser extent, the MR-MTBR/R’ and CT pools of Ts21 tau fragments (Fig. 1c, Fig. 1Sa). Whereas LTP inhibition by Ts21 iN secretomes was prevented by ID with Tau5 (Fig. 1d), partial ID with an antibody directed to the extreme CT of tau (aa404-441), Tau46, appeared ineffective (Fig. 1Sb). In contrast, ID with K9JA, a polyclonal antibody recognising a wide epitope including the MTBR and CT of tau (aa243-441), had similar effects to ID with Tau5. Thus, K9JA ID of Ts21 iN secretomes reduced CT tau by ~ 85%, with a modest effect on MR tau (~ 30%) when compared with Mock ID and fully abrogated the LTP deficit (Fig. 1Sc, d).

To investigate whether regions CT to the MTBR were present in synaptotoxic forms of tau, we probed the region of tau spanning the R’ and adjacent CT sequence using two novel monoclonal antibodies, Gen2B and Gen2A [32, 33]. Both Gen2B, which is directed at the R’ domain (aa369-381) and Gen2A (targeting R’ and the adjacent CT region, aa396-410), were very effective in ID of MR-MTBR/R’ tau in Ts21 iN secretomes (> 85% reduction in both cases, Fig. 1e,g). Importantly, the magnitude of LTP was indistinguishable from controls when animals were injected with either Gen2B ID (Fig. 1f) or Gen2A ID (Fig. 1h) secretomes. Complementing the ID approach, we co-injected some animals with Gen2A to neutralise tau fragments containing the CT sequence proximal to the MTBR. These animals maintained normal LTP after i.c.v administration of a mixture of Ts21 secretome and Gen2A mAb (Fig. 2S).

To further study the nature of Ts21 iN-derived synaptotoxic tau fragments, we used Ts21 secretomes that had been fractionated by SEC [28]. Figure 3Sa outlines the distribution of MR and CT tau fragments across the fractions determined using immunoassays, consistent with what was described by us previously [28]. Due to the low tau concentration in the fractionated Ts21 materials, no signal was detected in any of the SEC fractions with our custom MR-MTBR/R’ immunoassay (lower limit of quantification, LLOQ = 500 pg/ml). To investigate the distribution of MTBR-containing tau across fractions we employed semi-quantitative MS. MTBR peptide sequences were found especially in fractions 12–14 and 16–17 (Fig. 2a).

Among the fractions tested in electrophysiological experiments, fractions 15 and 16 significantly inhibited LTP when compared with vehicle-injected control animals (Fig. 2b). Although we had insufficient amounts of fraction 15 to characterise with MS, it is likely that the tau sequence in fraction 15 is very close to that in the two flanking fractions because the same MR and MTBR tau sequences (apart from MR aa 195–209, i.e. seven of eight sequences measured) were present in fraction 16, which had similar biological activity to fraction 15, and fraction 14, which was not tested in vivo (Fig. 2a). Like unfractionated Ts21 secretomes (Fig. 1d), ID of fraction 15 with the MR-directed mAb Tau5, but not one directed to the extreme CT of tau (Tau46), prevented the disruption of synaptic plasticity (Fig. 3Sb). Significantly, co-injection of fraction 15 with the Gen2A mAb, which recognises an epitope in the CT sequence proximal to R’, was also effective in preventing the inhibition of LTP (Fig. 2c).

Taken together, these findings implicate MTBR/R’-containing and related fragments in mediating the synaptic plasticity-disrupting actions of Ts21 iN secretomes. To test the hypothesis that such tau fragments are sufficient to mediate the synaptotoxicity of tau, we tested a recombinant protein encompassing tau_297-391_. This fragment of tau extends beyond the classical MTBR to include the R’ Gen2B epitope sequence and is the dominant species present in AD brain paired helical filaments [39]. Intriguingly, this fragment of tau robustly inhibited LTP (Fig. 2d).

### Anti-MTBR/R’ tau antibodies abrogate synaptotoxicity of AD brain soluble tau

Having established the ability of antibodies targeting R’ and sequences adjacent to the N- and C-termini of MTBR/R’ to prevent inhibition of LTP by Ts21 extracellular tau fragments, we evaluated the involvement of MTBR-related tau in synaptic plasticity disruption by tau-containing AD brain extracts. To this end, we injected synaptotoxic tau-containing aqueous brain extracts of two patients diagnosed with sporadic AD (referred to as AD1 and AD6) [40–42]. Western blotting (Fig. 3a) revealed that, apart from full-length tau, the two R’-directed Gen2 mAbs detected likely truncated tau fragments, some of which appeared to be recognised by both mAbs. Since the soluble extracts are prepared by homogenisation and most tau is intracellular, the relative contribution of brain ISF tau in the injectates is very small (< 1%) [20, 61].

Co-injection of either Gen2A or Gen2B mAbs with AD1 extract completely restored LTP (Fig. 3b). Separately, co-injection of Gen2B mAb with AD6 extract also abrogated the plasticity-disrupting activity of AD6 (Fig. 3c). These data indicate that the plasticity-disrupting activity of tau in AD brain extracts involves forms of tau which contain the R’ and adjacent CT sequences.

Finally, we wondered if tau fragments spanning MR to MTBR/R’ also mediated a persistent synaptotoxic effect of AD brain tau [42] (Fig. 4a). Remarkably, human tau was detectable in rat hippocampus 3 weeks after a single i.c.v. injection of AD1 extract (Fig. 4b and Fig. 4S). Furthermore, i.c.v. injection of Tau5 mAb just 15 min prior to HFS significantly reversed the LTP deficit in animals injected with AD1 extract 3 weeks earlier (Fig. 4c), consistent with an MR tau-dependence of the persistent LTP inhibition and our previous report using AD6 extract [42]. Importantly, administration of either Gen2A or Gen2B mAbs also rapidly reversed the LTP deficit in this model (Fig. 4d).

## Discussion

There is a rapidly growing clinical interest in exploring ways of both treating AD and other tauopathies with MTBR tau-directed therapies and also monitoring disease status with MTBR tau-based fluid biomarkers. Apart from being a major component of tau tangles, MTBR/R’ is also present in a soluble form in AD brain [25, 55] and human CSF [23–26] and plasma [27]. Understanding the involvement of MTBR tau in disease mechanisms should greatly inform these clinical developments. Here, we provide convincing evidence that forms of tau which appear to span the MR and MTBR/R’ mediate the synaptotoxicity of patient-derived tau. Either co-injection or ID with two novel MTBR/R’-directed antibodies prevented the acute inhibition of hippocampal LTP by two different AD-relevant preparations: Down syndrome Ts21 iN secretomes and aqueous extracts of AD brain. Furthermore, in vivo administration of these agents rapidly reversed the persistent disruption of synaptic plasticity by the brain extracts. Taken together with their potential involvement in the spread of pathology [49], our findings lend new mechanistic support for a focus on soluble MTBR/R’-containing tau species for future therapy and diagnostics.

Our present discovery that two anti-tau antibodies targeting MTBR/R’ and the adjacent CT region, together with our previous reports with the MR-directed antibody Tau5 [28, 40–42], abrogated the ability of both of the patient-derived tau preparations to inhibit LTP underlines the importance of tau species containing these regions in mediating synaptotoxicity. In the case of Ts21 iNs, a wide range of putatively extracellular truncated tau species are present in the secretome (Fig. 1S and Hu et al. [28]). In the latter study [28] secretomes from familial AD PS1 L113_I114insT iNs had a similar tau fragment profile but inhibited LTP in an Aß- not tau-dependent manner. In the present study, the Ts21 secretomes were found to contain significant amounts of fragments that included both the MR and MTBR/R’ sequences recognised by Tau5 and Gen2B, respectively. Suggestively, these fragments, detected using a novel immunoassay, were markedly reduced by ID with these mAbs (Fig. 1), strongly implicating fragments that encompass both MR and MTBR/R’ as mediators of the inhibition of LTP by Ts21 iN secretomes. The ability of K9JA, targeting both the MTBR and CT, to prevent the inhibition of LTP by Ts21 iN secretomes, unlike Tau46, which targets the extreme CT, is consistent with this conclusion. However, the efficiency of ID with Tau46 was relatively weak. In the case of the AD aqueous extracts, it is not possible to differentiate if synaptotoxicity is mediated by full-length tau or one or more MTBR/R’-containing fragments, or whether the active species are derived from within neurons or extracellularly. Nonetheless, our data from AD brain extracts confirm the requirement of the MR and MTBR/R’ domains for synaptotoxic activity. In view of the protective activity of Gen2A (Figs. 1–4; Fig. 2S), the rogue tau species also likely include peptides incorporating the CT sequence flanking MTBR/R’. The ability of recombinant tau_297-391_ to replicate the synaptic plasticity-disrupting action of patient-derived tau supports the hypothesis that peptides containing a core MTBR/R’ tau sequence are necessary and sufficient to mediate the synaptotoxicity of tau. We and others previously reported that recombinant preparations of full-length tau needed to be pre-aggregated to inhibit LTP [14, 40–42], whereas tau_297-391_ appears to be a very potent synaptotoxin without deliberate aggregation. Although unlikely, we cannot rule out the possibility that this aggregation-prone tau species may form low levels of oligomers prior to or soon after injection into live rat brain*.* The most parsimonious explanation for our results is that fragments spanning from the MR to just CT of MTBR/R’ (218–410) define the synaptotoxic forms of tau in the specimens studied. It is also possible that at least two small fragments of tau, one MR and the other MTBR/R’-containing, are acting in concert to cause synaptic plasticity impairment. Interestingly, both MR and MTBR-containing fragments were found in the potent SEC fraction 16 of Ts21 iN secretomes (Fig. 2a). Since AD aqueous brain extracts contain a very wide range of intracellular and extracellular post-translationally modified tau species, it is likely that the nature of the synaptotoxic culprits in these preparations is even more complex than those in the Ts21 iN secretomes.

We confirmed and extended our previous reports that patient-derived synaptotoxic tau-containing brain extracts potently inhibit LTP for several weeks in vivo [41, 42]. The rapid reversal of the deficit by the two anti-MTBR/R’ antibodies using 15-min i.c.v. treatment, 3 weeks after AD1 extract exposure, indicates the prolonged presence of readily accessible synaptotoxic AD tau. Consistent with this hypothesis, human tau was detected in the hippocampus of these animals at this time (Fig. 4a, Fig. 4S). Given that only a brief exposure was needed for the antibodies to exert their protective action, it is likely that they directly bound and thereby neutralised the synaptotoxic MTBR/R’-containing tau species.

Although it is expected that the exogenously applied synaptotoxic tau and anti-tau antibodies act extracellularly under the current experimental conditions, we cannot rule out a role for intracellular actions and interactions. Tau can readily cross plasma membranes and several transporters that carry tau are known [10, 13, 22, 34, 44, 47]. The inhibition of LTP by exogenous tau has been reported to require amyloid precursor protein (APP)-dependent tau uptake [45] and direct injection of tau postsynaptically potently inhibits LTP [21]. Toxic tau species have multiple intracellular targets, including microtubules, endosomes, lysosomes and proteasome systems [5, 6, 10, 44, 48], all of which are important for normal synaptic function and plasticity. Similarly, some anti-tau antibodies can successfully interact with tau intracellularly [9, 17, 54]. Indeed, amelioration of pathological tau’s deleterious action on proteostasis by anti-tau antibodies is at least partly mediated by tripartite motif-containing 21 (TRIM21), a cytosolic antibody Fc receptor and E3 ligase [15, 35, 37]. Moreover, certain intracellularly generated anti-tau modified antibody fragments (intrabodies) have been reported to be more efficacious at clearing pathogenic tau than exogenously applied antibodies [16]. In the case of Gen2A and Gen2B, relatively limited amounts enter neurons in the absence of tau [32, 33]. However, when bound to tau, Gen2A is a substrate of tau uptake mechanisms, whereas Gen2B inhibits tau transport [32, 33]. Clearly, given the abundance of intracellular MTBR-containing pathological tau in AD brain, developing MTBR-targeting tau antibodies that can cross the plasma membrane is potentially advantageous [54].

In conclusion, our discovery that MTBR/R’-containing and related fragments are potent mediators of the synaptic plasticity-disrupting actions of patient-derived soluble synaptotoxic tau provides a biological basis for interpreting ongoing biomarker-based clinical trials in AD immunotherapy.

## Supplementary Information

Below is the link to the electronic supplementary material.

Supplementary file1 (PDF 1430 KB)

## Data Availability

Data are provided within the manuscript or supplementary information files. The original data are available upon reasonable request.
