# Supplementary material for: Synaptotoxic effects of extracellular tau are mediated by its microtubule-binding region
Source: Acta Neuropathol. 2025 Jun 2;149(1):56. doi: 10.1007/s00401-025-02897-0 (PMC12130085; doi:10.1007/s00401-025-02897-0)
Supplement: Supplementary file 1 — Supplementary file1 (PDF 1430 KB) [file 401_2025_2897_MOESM1_ESM.pdf]

Supplementary information

Synaptotoxic effects of extracellular tau are mediated by its microtubule-binding region

Tomas Ondrejcak[1], Neng-Wei Hu[1,2], Emily Coode[3], Tom Campbell[3], Grant T. Corbett[4], Ivan Doykov[5], Kevin Mills[5], Dominic M. Walsh[4], Frederick J. Livesey[3,6], Michael J. Rowan[1] and Igor Klyubin[1]

Author affiliations:

1 Department of Pharmacology and Therapeutics, School of Medicine, and Institute of Neuroscience, Trinity College, Dublin 2, Ireland

2 Department of Physiology and Neurobiology, School of Basic Medical Sciences, Zhengzhou University, 100 Science Avenue, Zhengzhou 450001, China

3 Talisman Therapeutics, Babraham Research Campus, Cambridge, CB22 3AT, UK

4 Laboratory for Neurodegenerative Research, Ann Romney Center for Neurologic Diseases, Brigham and Women's Hospital and Harvard Medical School, Boston, MA, USA.

5 Translational Mass Spectrometry Research Group, University College London Institute of Child Health and Great Ormond Street Hospital, London, UK.

6 Gen2 Neuroscience, Babraham Research Campus, Cambridge, CB22 3AT, UK

Correspondence to: Michael J. Rowan or Igor Klyubin

Full address: Pharmacology & Therapeutics, Watts Building, Trinity College Dublin, Dublin 2, Ireland. E-mail: mrowan@tcd.ie or klyubini@tcd.ie

DOI: 10.1007/500401-025-02897-0

Supplementary Material

(1) Figures 1-5S

(2) Tables 1-2S

(3) Supplementary Materials and Methods

(4) Supplementary References

Supplementary Figures

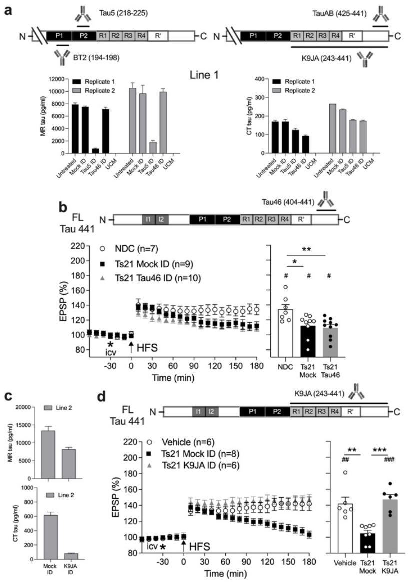

Figure 1S. **Effect of anti-tau antibodies directed to the MTBR/CT and extreme CT on the synaptotoxicity of patient-derived extracellular tau** (**a**) Insets show antibodies used to capture/detect mid-region (MR) and C-terminal (CT)-containing tau fragments in sandwich ELISAs. Effect of ID with MR-directed Tau5 and extreme CT-directed Tau46 anti-tau mAbs on levels of MR and CT-containing tau fragments. Secretomes from Line 1 of two lines of Ts21 iNs or unconditioned media (UCM) were assayed twice using Tau5-BT2 and K9JA-TauAB sandwich ELISAs. Tau5 ID reduced MR-containing tau fragments by ~95% and CT pool by ~30%. Tau46 ID reduced the CT pool by ~30-40%, with little detectible effect on MR tau. In contrast to Tau46 (**b**), K9JA ID lowered both CT and, to a lesser extent, MR tau levels (**c**) and prevented inhibition of hippocampal LTP under urethane anaesthesia (**d**). Left-hand panel shows the time course of LTP. Summary bar chart of LTP magnitude during the last 10 min is in the right-hand panel. Values are mean ± SEM. $^{\#}p < 0.05$, $^{\#\#\#\#}p < 0.0001$ compared with pre-HFS, paired *t*-test; $^{*}p < 0.05$, $^{**}p < 0.01$, $^{***}p < 0.001$, one-way ANOVA followed by Bonferroni's multiple-comparison tests.

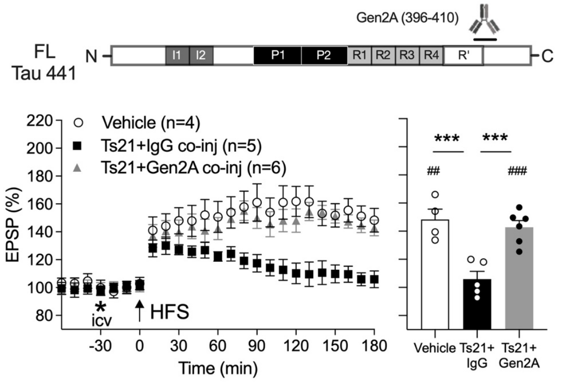

Figure 2S. **Co-injection of an antibody directed C-terminal of the R' domain (Gen2A) abrogates Ts21 iN secretome-induced LTP deficit.** I.c.v. injection of Gen2A (2.5 µg)-containing Ts21 iN secretome failed to inhibit LTP, unlike co-injection of an isotype control IgG (2.5 µg). Left-hand panel shows the time course of LTP. Summary bar chart of LTP magnitude during the last 10 min is in the right-hand panel. Values are mean ± SEM. $^{\#\#}$p < 0.01, $^{\#\#\#}$p < 0.001 compared with pre-HFS, paired *t*-test; ***p < 0.001, one-way ANOVA followed by Bonferroni's multiple-comparison tests.

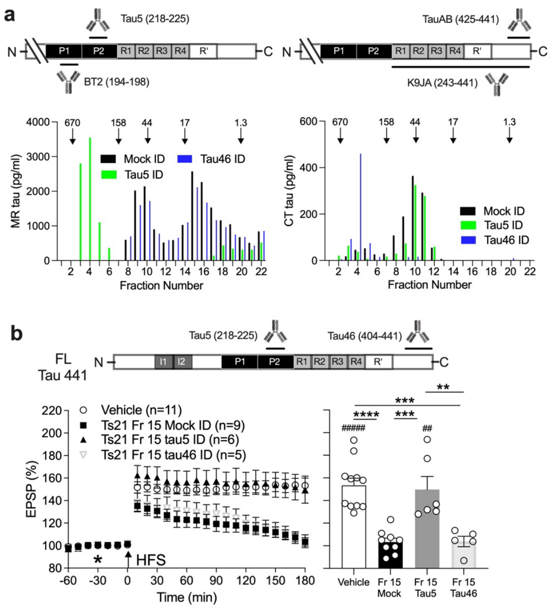

Figure 3S. **Abrogation of the inhibition of LTP by a biologically active SEC fraction of Ts21 iN secretome by immunodepletion (ID) with mid-region-directed antibody** (**a**) Effect of ID with mid-region (MR) directed Tau5 and C-terminally (CT) directed Tau46 anti-tau mAbs on MR and CT tau fragments. The concentration of MR and CT-containing tau in the different SEC fractions were assayed using Tau5-BT2 and K9JA-TauAB sandwich ELISAs, respectively. The elution of globular protein standards (the molecular weight of which are given in kDa) is indicated by downward pointing arrows on the top of the chromatogram. (**b**) Whereas ID of a biologically active fraction of Ts21 secretomes, fraction 15 (Ts21 Fr 15 tau5 ID) prevented inhibition of LTP, ID with Tau 46 (Ts21 Fr 15 tau46 ID did not, when compared with mock ID of fraction 15 (Ts21 Fr 15 Mock ID) or vehicle-injected rats (Vehicle). Left-hand panel shows the time course of LTP. Summary bar chart of LTP magnitude during the last 10 min is in the right-hand panel. Values are mean ± SEM. $^{\#\#}$p < 0.01, $^{\#\#\#\#}$p < 0.0001 compared with pre-HFS, paired *t*-test; **p < 0.01, ***p < 0.001, ****p < 0.0001, one-way ANOVA followed by Bonferroni's multiple-comparison tests.

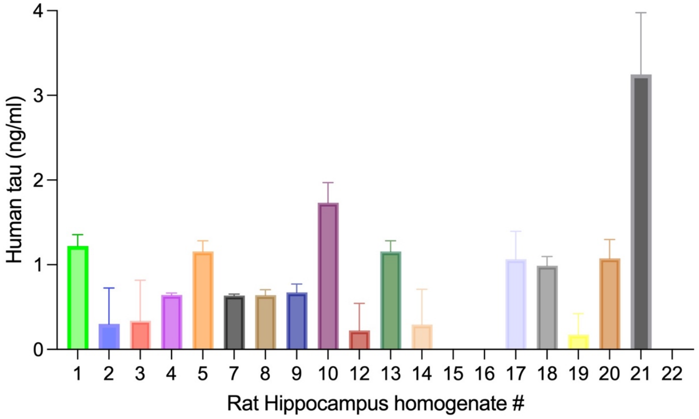

Figure 4S. **Concentration of human tau in individual rat hippocampal homogenates after intracerebroventricular (i.c.v.) injection of Alzheimer's disease soluble brain extract** Three weeks after animals # 1-20 received a single i.c.v. injection of AD1 aqueous extract (either Mock ID, # 1-5 and 7-10, or AW7 ID, # 12-20) under recovery anaesthesia their brains were removed under non-recovery anaesthesia and the concentration of human tau assayed in hippocampal homogenates. We could not analyse homogenates from rats # 6 and # 11 because of technical issues. Homogenate # 21 is a pooled sample from three rats acutely injected i.c.v. with the same volume of AD1 Mock ID extract 30 min previously. Animal # 22 received a single intrahippocampal acute sham injection. LLoQ was 0.03125 ng/ml.

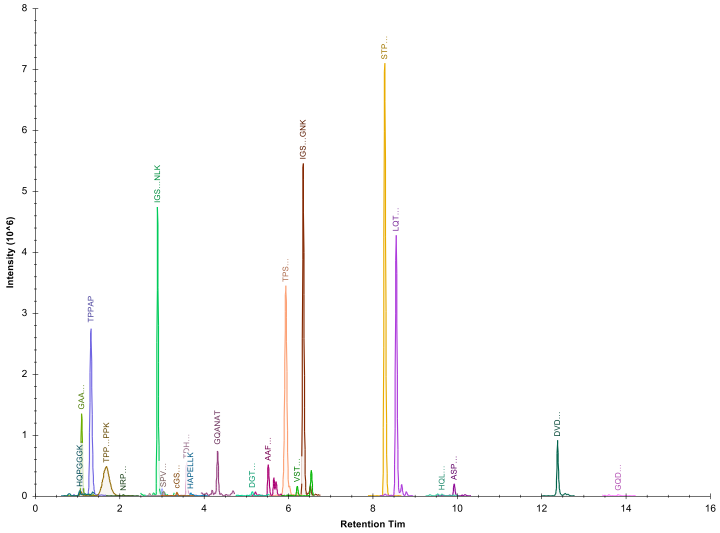

Figure 5S **Example of peptide chromatography- Pooled quality control (QC) sample**

Supplementary Tables

Table 1S **Primary antibodies and their antigens, dilutions and sources.**

**Antibody**	**Clonality**	**Antigen/Epitope**	**Dilution**	**Source**	**Reference**
46-4	Monoclonal	Anti-HIV	1:100 (immunoprecipitation, IP)	ATCC	Wang et al. [10]
BT2	Monoclonal	Tau 194-198	2.5 µg/ml (ELISA)	ThermoFisher	Mercken et al. [6]
Gen2A	Monoclonal	Tau 396-410	1:100 (IP);<br>2.5 µg (i.c.v.)	Gen2 Neuroscience	In-house antibody. [3]
Gen2B	Monoclonal	Tau 369-381	1:100 (IP);<br>4 µg/ml (MSD);<br>1:1000 (WB);<br>2.5 µg (i.c.v.)	Gen2 Neuroscience	In-house antibody. [4]
IgG1κ	Monoclonal	N/A	2.5 µg (i.c.v.)	BioLegend	Ondrejcak et al. [8]
IgG rabbit	Monoclonal	N/A	1:100 (IP);<br>2.5 µg (i.c.v.)	eBioscience	Bode et al. [1]
K9JA	Polyclonal	Tau 243-441	1:100 (IP);<br>2.5 µg/ml (ELISA)	Dako	Wang et al. [10]
Tau46	Monoclonal	Tau 404-441	1:100 (IP)	BioLegend	Meredith et al. [7]
Tau5	Monoclonal	Tau 218-225	1:100 (IP);<br>2.5 µg/ml (ELISA);<br>2.5 µg (i.c.v.)	BioLegend	Porzig et al. [9]
			2 µg/ml (MSD)	ThermoFisher	
TauAB	Monoclonal	Tau 425-441	2.5 µg/ml (ELISA)	MedImmune	Hu et al. [2]

Table 2S **Table of transitions used in the targeted assay**

**Peptide Sequence**	**Precursor Mz**	**Precursor Charge**	**Collision Energy**	**Product Mz**	**Product Charge**	**Fragment Ion**
STPTAEDVTAPLVDEGAPGK	652.325	3	22.2	982.5204	1	y10
STPTAEDVTAPLVDEGAPGK	652.325	3	22.2	673.3151	1	y7
STPTAEDVTAPLVDEGAPGK	652.325	3	22.2	491.7638	2	y10
HAPELLK	404.2398	2	13.9	670.4134	1	y6
HAPELLK	404.2398	2	13.9	599.3763	1	y5
HAPELLK	404.2398	2	13.9	435.1987	1	b4
HQLLGDLHQEGPPLK	561.3055	3	18.9	768.425	1	y7
HQLLGDLHQEGPPLK	561.3055	3	18.9	453.2456	2	y8
HQLLGDLHQEGPPLK	561.3055	3	18.9	664.3413	1	b6
DVDESSPQDSPPSK	496.5547	3	16.5	515.2824	1	y5
DVDESSPQDSPPSK	496.5547	3	16.5	428.2504	1	y4
DVDESSPQDSPPSK	496.5547	3	16.5	428.214	2	y8
ASPAQDGRPPQTAAR	761.8897	2	27.1	740.405	1	y7
ASPAQDGRPPQTAAR	761.8897	2	27.1	682.8551	2	y13
ASPAQDGRPPQTAAR	761.8897	2	27.1	477.2674	2	y9
VSTEIPASEPDGPSVGR	566.6162	3	19.1	784.3948	1	y8
VSTEIPASEPDGPSVGR	566.6162	3	19.1	572.3151	1	y6
VSTEIPASEPDGPSVGR	566.6162	3	19.1	392.701	2	y8
GQDAPLEFTFHVEITPNVQK	757.3866	3	25.9	686.3832	1	y6
GQDAPLEFTFHVEITPNVQK	757.3866	3	25.9	585.3355	1	y5
GQDAPLEFTFHVEITPNVQK	757.3866	3	25.9	950.0042	2	y16
AAFPGAPGEGPEAR	442.8861	3	14.6	715.3369	1	y7
AAFPGAPGEGPEAR	442.8861	3	14.6	529.2729	1	y5
AAFPGAPGEGPEAR	442.8861	3	14.6	472.2514	1	y4
DGTGSDDK	397.6618	2	13.6	679.2893	1	y7
DGTGSDDK	397.6618	2	13.6	521.2202	1	y5
DGTGSDDK	397.6618	2	13.6	464.1987	1	y4
NRPCLSPK	486.2582	2	16.9	528.2347	1	b4
NRPCLSPK	486.2582	2	16.9	641.3188	1	b5
NRPCLSPK	486.2582	2	16.9	728.3508	1	b6
GAAPPGQK	363.2007	2	12.4	597.3355	1	y6
GAAPPGQK	363.2007	2	12.4	526.2984	1	y5
GAAPPGQK	363.2007	2	12.4	263.6528	2	y5
GQANATR	359.1856	2	12.2	660.3424	1	y6
GQANATR	359.1856	2	12.2	532.2838	1	y5
GQANATR	359.1856	2	12.2	461.2467	1	y4
TPPAPK	305.6816	2	10.2	412.2554	1	y4
TPPAPK	305.6816	2	10.2	255.1577	2	y5
TPPAPK	305.6816	2	10.2	206.6314	2	y4
TPPSSGEPPK	498.7535	2	17.4	798.3992	1	y8

TPPSSGEPPK	498.7535	2	17.4	448.2296	2	y9
TPPSSGEPPK	498.7535	2	17.4	399.7032	2	y8
SGYSSPGSPGTPGSR	697.3208	2	24.7	912.4534	1	y10
SGYSSPGSPGTPGSR	697.3208	2	24.7	671.3471	1	y7
SGYSSPGSPGTPGSR	697.3208	2	24.7	456.7303	2	y10
TPSLPTPPTR	533.7982	2	18.7	868.4887	1	y8
TPSLPTPPTR	533.7982	2	18.7	668.3726	1	y6
TPSLPTPPTR	533.7982	2	18.7	286.1636	2	y5
LQTAPVPMPDLK	655.3629	2	23.2	1068.5758	1	y10
LQTAPVPMPDLK	655.3629	2	23.2	896.491	1	y8
LQTAPVPMPDLK	655.3629	2	23.2	700.3698	1	y6
IGSTENLK	431.2375	2	14.9	748.3836	1	y7
IGSTENLK	431.2375	2	14.9	691.3621	1	y6
IGSTENLK	431.2375	2	14.9	604.3301	1	y5
HQPGGGK	340.6774	2	11.5	543.2885	1	y6
HQPGGGK	340.6774	2	11.5	415.23	1	y5
HQPGGGK	340.6774	2	11.5	318.1772	1	y4
CGSLGNIHHKPGGGQVEVK	658.6707	3	22.4	870.468	1	y9
CGSLGNIHHKPGGGQVEVK	658.6707	3	22.4	878.9763	2	y17
CGSLGNIHHKPGGGQVEVK	658.6707	3	22.4	778.9182	2	y15
IGSLDNITHVPGGGNK	526.946	3	17.6	529.2729	1	y6
IGSLDNITHVPGGGNK	526.946	3	17.6	733.3733	2	y15
IGSLDNITHVPGGGNK	526.946	3	17.6	704.8626	2	y14
TDHGAEIVYK	378.1926	3	12.3	522.3286	1	y4
TDHGAEIVYK	378.1926	3	12.3	482.1994	1	b5
TDHGAEIVYK	378.1926	3	12.3	611.242	1	b6
SPVVSGDTSPR	551.2804	2	19.3	917.4687	1	y9
SPVVSGDTSPR	551.2804	2	19.3	818.4003	1	y8
SPVVSGDTSPR	551.2804	2	19.3	719.3319	1	y7

Supplementary Materials and Methods

Mass spectrometry

Assay Development

The full sequence of the tau protein was obtained from UNIPROT (August 2023), and unique tryptic peptides were identified. These peptides were subsequently imported into Skyline (MacCoss Lab Software, 23.1.0.268) [5], where, in conjunction with Prosit (www.proteometools.org), an *in silico* spectral library was generated for each peptide across different precursor charge states (+2, +3, and +4). A normalised collision energy (NCE) of 31, which was experimentally established for this instrument, was utilised to create the spectral library. The fragment selection for the specified charge states was refined by filtering to retain the 12 most intense fragments predicted by the *in silico* spectral library. This process facilitated the exportation of multiple acquisition methods to MassLynx (Waters), each configured with a minimum dwell time per transition of 10 µs. Chromatographic conditions and injection volumes are described in subsequent sections. A tryptic peptide digest of human recombinant tau352 with a C-terminal His tag (Abcam, ab316441) was utilised for method development, in addition to the pooled quality control (QC) sample (described in the Pre-analysis Sample Preparation section and Figure 5S). Following data acquisition (Xevo TQ-XS Triple Quadrupole Mass Spectrometer and ACQUITY UPLC I-Class PLUS System, Waters), raw data were imported into Skyline for evaluation, and peptide identity was confirmed through spectral library matching (dot product score cut-off of 0.75). The selection of the predominant precursor charges and fragments was based on the intensity and chromatographic performance of the pooled QC sample. This was followed by an additional round of method export, focusing on collision energy optimisation for selected precursors and fragments (see Table 2S). Utilising the experimental data, a final method with three fragments per peptide was developed for the scheduled acquisition of each peptide within a 0.8-minute window to minimise overlap and enhance sensitivity.

Sample Preparation and Digestion Protocol

Lyophilised fractions were re-suspended in 900 µL of dH_20 and a 450 µL aliquot from each fraction was transferred into 1.5 mL microtubes and subjected to lyophilisation overnight

again. Subsequently, the dried samples were resuspended in 20 µL of digestion buffer (6M urea, 2M thiourea, and 2% ASB-14 in 200 mM Tris-HCl, pH 8.0) and incubated at room temperature for 1 h with agitation to facilitate protein denaturation. Disulfide bond reduction was achieved through the addition of 3 µL of 169 mM dithiothreitol (DTT) and incubation for 1 h at room temperature with shaking. Alkylation was performed by adding 6 µL 169 mM iodoacetamide (IAA) and incubating for 45 min in the dark at room temperature. To facilitate efficient trypsin digestion, the mixture was diluted with 165 µL of Milli-Q water to reduce the urea concentration below 1M. Trypsin Gold (Promega, V5280) was prepared at a concentration of 0.1 µg/µL in 200 mM Tris-HCl (pH 8.0). Digestion was initiated by adding 10 µL trypsin solution (1 µg) to each sample, followed by brief vortexing. The samples were then subjected to digestion for 16 h at 37°C with agitation in a thermomixer (Eppendorf, 5382000031). The reaction was terminated by the addition of 200 µL 0.2% trifluoroacetic acid (TFA).

Peptide Cleanup via Solid Phase Extraction (SPE)

Following trypsin digestion, peptide mixtures underwent purification via solid-phase extraction (SPE) to attain high purity and concentration, which is essential for subsequent mass spectrometric analysis. The SPE cleanup procedure commenced with the preconditioning of Biotage SPE cartridges, which were pre-wetted twice with 1 mL of 60% acetonitrile (ACN) in 0.1% trifluoroacetic acid (TFA), ensuring adequate preparation for peptide binding. This was followed by an equilibration step in which cartridges were treated twice with 1 mL of 0.1% TFA to optimise the binding environment for the peptides. Acidified peptide samples were then loaded onto equilibrated cartridges and allowed to elute under gravity, a process designed to facilitate effective peptide binding. To further purify the captured peptides, the cartridges were washed twice with 0.1% TFA to eliminate residual impurities. Peptides were subsequently eluted into new 1.5 mL microtubes using two sequential aliquots of 500 µL of 60% ACN in 0.1% TFA, ensuring peptide recovery. The eluted peptides were then desiccated using a rotational evaporator under vacuum at room temperature for 7.5 hours, resulting in dry peptide samples that were stored at − 20º C until analysis.

Pre-analysis Sample Preparation and Chromatographic Conditions

The dried peptide digests were reconstituted in 100 µL of 5% ACN and 0.1% TFA. The peptide concentration was determined using the Pierce Colorimetric Peptide assay. Each sample was diluted to a final concentration of 100 ng/µL, and a pooled QC sample comprising 30 µL of each digest was prepared. Chromatographic separation was achieved on a 10 cm Acquity Premier column (2.1 mm diameter, 1.7 µm particle size) (BEH C18, 130Å, Waters, 186009658), with the column maintained at 60°C. The injection volume was 5 µL, equivalent to 500 ng of peptides on the column. The initial mobile phase conditions were 95% A (0.1% formic acid in water) and 5% B (0.1% formic acid in acetonitrile), maintained for 1 min before applying a gradient over 13.4 minutes to reach 35% B, followed by a ramp to 100% B for 1 min, and sustained for an additional 2 min. The system was subsequently returned to the initial conditions for column equilibration with a total run time of 20 min per injection.

Instrument Parameters

The instrument was operated in positive ion mode with a capillary voltage of 2.8 kV and a cone voltage maintained at 35 V. The source and desolvation temperatures were set at 150°C and 500°C, respectively, with desolvation and cone gas flows of 700 L/h and 150 L/h, respectively.

Final Method and Analysis

Samples were analysed utilising the final method, with pooled samples injected subsequent to every third sample to monitor instrument performance. Data integration was conducted using Skyline, and the results were exported to a CSV file for further analysis in Excel.

Supplementary References

1 Bode K, MacDonald T, Stewart T, Mendez B, Cai EP, Morrow N, Lee YC, Yi P, Kissler S (2023) Protective renalase deficiency in beta-cells shapes immune metabolism and function in autoimmune diabetes. Diabetes 72: 1127-1143 Doi https://doi.org/10.2337/db23-0030

2 Hu NW, Corbett GT, Moore S, Klyubin I, O'Malley TT, Walsh DM, Livesey FJ, Rowan MJ (2018) Extracellular forms of Aβ and tau from iPSC models of Alzheimer's disease disrupt synaptic plasticity. Cell Rep 23: 1932-1938 Doi https://doi.org/10.1016/j.celrep.2018.04.040
3 Livesey FJ, Jones C (2021) Tau epitope and binding molecules: Gen2A World Patent no. WO2021005019A1. https://worldwideespacenetcom/patent/search/family/067623123/publication/WO2021005019A1?q=pn%3DWO2021005019A1:
4 Livesey FJ, Jones C (2020) Tau epitope and binding molecules: Gen2B World Patent no. WO2020260722A1. https://worldwideespacenetcom/patent/search/family/067540072/publication/WO2020260722A1?q=pn%3DWO2020260722A1:
5 MacLean B, Tomazela DM, Shulman N, Chambers M, Finney GL, Frewen B, Kern R, Tabb DL, Liebler DC, MacCoss MJ (2010) Skyline: an open source document editor for creating and analyzing targeted proteomics experiments. Bioinformatics 26: 966-968 Doi https://doi.org/10.1093/bioinformatics/btq054
6 Mercken M, Vandermeeren M, Lubke U, Six J, Boons J, Van de Voorde A, Martin JJ, Gheuens J (1992) Monoclonal antibodies with selective specificity for Alzheimer Tau are directed against phosphatase-sensitive epitopes. Acta Neuropathol 84: 265-272 Doi https://doi.org/10.1007/BF00227819
7 Meredith JE, Jr., Sankaranarayanan S, Guss V, Lanzetti AJ, Berisha F, Neely RJ, Slemmon JR, Portelius E, Zetterberg H, Blennow Ket al (2013) Characterization of novel CSF Tau and ptau biomarkers for Alzheimer's disease. PLoS One 8: e76523 Doi https://doi.org/10.1371/journal.pone.0076523
8 Ondrejcak T, Klyubin I, Hu NW, O'Malley TT, Corbett GT, Winters R, Perkinton MS, Billinton A, Prenderville JA, Walsh DMet al (2023) Tau and Amyloid β protein in patient-derived aqueous brain extracts act concomitantly to disrupt long-term potentiation *in vivo*. J Neurosci 43: 5870-5879 Doi https://doi.org/10.1523/JNEUROSCI.0082-23.2023
9 Porzig R, Singer D, Hoffmann R (2007) Epitope mapping of mAbs AT8 and Tau5 directed against hyperphosphorylated regions of the human tau protein. Biochem Biophys Res Commun 358: 644-649 Doi https://doi.org/10.1016/j.bbrc.2007.04.187
10 Wang YP, Biernat J, Pickhardt M, Mandelkow E, Mandelkow EM (2007) Stepwise proteolysis liberates tau fragments that nucleate the Alzheimer-like aggregation of full-length tau in a neuronal cell model. Proc Natl Acad Sci U S A 104: 10252-10257 Doi https://doi.org/10.1073/pnas.0703676104
